# First person – Ralitsa Madsen

**DOI:** 10.1242/dmm.048939

**Published:** 2021-03-11

## Abstract

First Person is a series of interviews with the first authors of a selection of papers published in Disease Models & Mechanisms, helping early-career researchers promote themselves alongside their papers. Ralitsa Madsen is first author on ‘NODAL/TGFβ signalling mediates the self-sustained stemness induced by *PIK3CA^H1047R^* homozygosity in pluripotent stem cells’, published in DMM. Ralitsa conducted the research described in this article while a member of Prof. Robert Semple's lab, initially as a PhD student at the Wellcome Trust-Medical Research Council Institute of Metabolic Science-Metabolic Research Laboratories at Addenbrooke's Hospital, University of Cambridge, UK, and then as a Postdoctoral Research Fellow at the University of Edinburgh, UK. She is now a Sir Henry Wellcome Postdoctoral Fellow in the lab of Prof. Bart Vanhaesebroeck at University College London Cancer Institute, London, UK, investigating the cellular context-dependent PI3K signalling code, and its reprogramming in human disorders such as cancer and benign overgrowth.


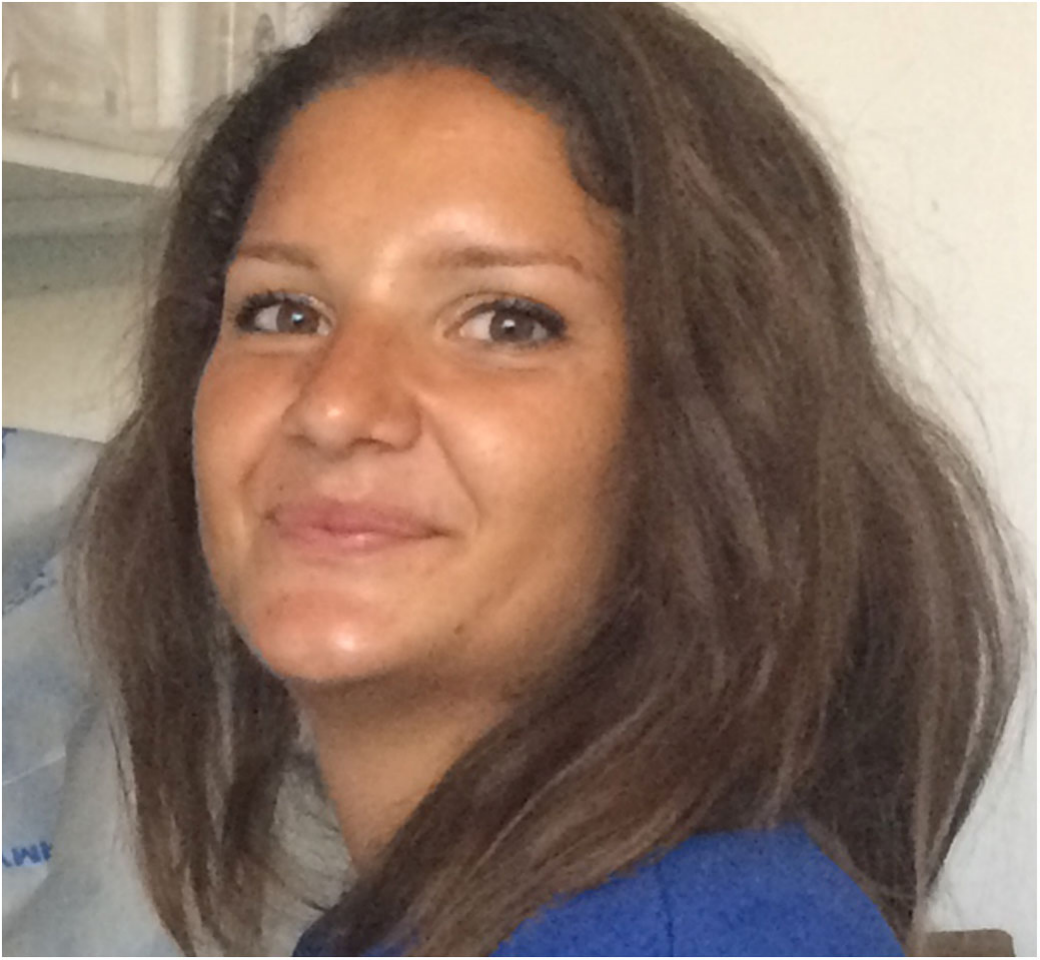


**Ralitsa Madsen**

**How would you explain the main findings of your paper to non-scientific family and friends?**

Many human diseases have their origins in faults within the molecular circuits that govern our cells. Such faults may be caused by DNA letter changes, also known as mutations. Depending on context, a mutation may give rise to distinct diseases. The mutation (*PIK3CA^H1047R^*) studied in this work is one such example. When acquired before birth, it is associated with benign overgrowth. Yet, it is also one of the most common mutations in adult human cancers. In contrast to overgrowth disorders, in which the *PIK3CA^H1047R^* mutation exists in a single copy within a cell, we recently discovered that cancers may have more than one mutant copy, leading to additional changes in the cellular circuitry. Using human pluripotent stem cells that model human developmental, as well as cancer processes, we now provide the first in-depth characterisation of the molecular circuit faults triggered by different doses (or mutant copies) of *PIK3CA^H1047R^*. We discovered a remarkable cellular robustness to the presence of a single copy of *PIK3CA^H1047R^*, in contrast to the major ‘rewiring’ caused by two *PIK3CA^H1047R^* copies. We also show that fixing this rewired system requires a fundamentally different strategy to the one typically considered in the context of disease-causing *PIK3CA* mutations.

**What are the potential implications of these results for your field of research?**

Our systematic profiling of the circuit faults caused by *PIK3CA^H1047R^* in human pluripotent stem cells provides the field with a comprehensive resource that will aid further research into mechanisms and treatments of *PIK3CA*-related disorders. Furthermore, our work emphasises the importance of carefully constructed and characterised cell models for *PIK3CA*-related disorders. We also hope that the insights that we offer in this context will promote improved standardisation and reproducibility in future attempts to develop complementary model systems.

**What are the main advantages and drawbacks of the model system you have used as it relates to the disease you are investigating?**

A key advantage of our model system is its versatility and disease relevance. Given their developmental origin, overgrowth disorders caused by *PIK3CA* mutations require model systems that can mimic key aspects of human development. To our knowledge, these are the first human pluripotent stem cell models with *PIK3CA* mutations. They have a diploid genome and are naturally immortal, which gives them characteristics of both ‘normal’ as well as cancer cells. They also have the additional advantage of being developed on an isogenic background, thus eliminating variability caused by different cellular genomes.

Yet, it is also a key limitation of our work that we do not have an expanded series of isogenic lines across a range of genetic backgrounds. Unfortunately, human pluripotent stem cells are highly demanding and expensive to keep in culture. For reproducible results, one also has to be extremely consistent with respect to cell culture maintenance in order to eliminate spontaneous differentiation and experimental variability. This makes it very challenging to use these cells on a routine basis. We hope that the community will complement our work by engineering additional stem cell models with differences in *PIK3CA* mutant dose so that we can assess the reproducibility of the observed dose-dependent effects across genetic backgrounds.

**What has surprised you the most while conducting your research?**

While conducting this research, what surprised us the most was the incredible robustness of human cells, such that a disease-causing genetic defect, like heterozygosity for *PIK3CA^H1047R^*, only leads to subtle cellular changes in a model system like ours. This is despite the fact that the same defect is associated with debilitating overgrowth disorders, collectively referred to as the *PIK3CA-*related overgrowth spectrum (PROS). It speaks to the power of adaptive responses within cells, there to safeguard the system against major internal and external perturbations. Yet, over the life-course of an individual, even subtle cellular changes can lead to severe human disease – PROS being a case in point. We must therefore remember the power of direct observation of the actual human ‘experiment’ conducted by nature (the disease itself), and correspondingly avoid overfitting observations from simplified experimental systems.

**Describe what you think is the most significant challenge impacting your research at this time and how will this be addressed over the next 10 years?**

A key challenge is knowing what the ‘right’ model system really is when it comes to *PIK3CA*-related diseases, be it overgrowth or cancer. Context, including cell lineage, is so important, yet we are far from an understanding of how exactly it may shape the consequences of *PIK3CA* mutations. The future looks promising though. Technological advances, such as CRISPR-based gene editing and single-cell-based assays, will likely help us shed light on the context-dependent effects of *PIK3CA* mutations. Long term, this will hopefully help us to develop tailored therapies for individuals suffering from *PIK3CA*-related diseases.

**Figure:**
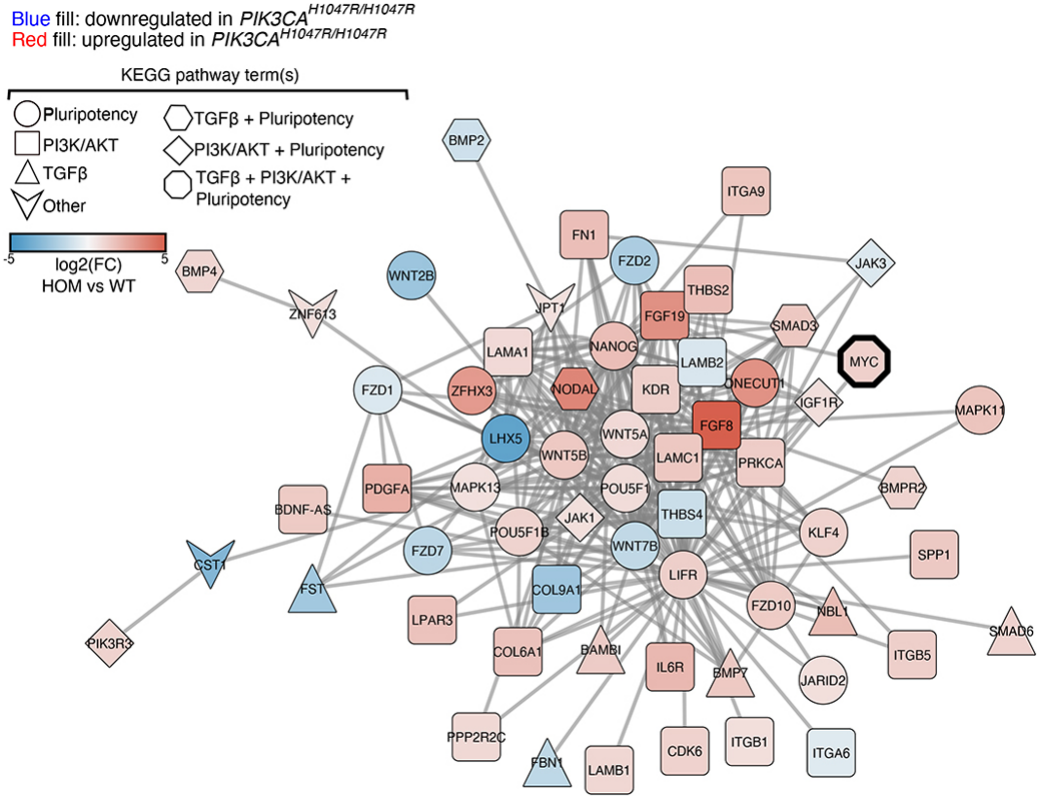
**Multi-omic analyses of human pluripotent stem cells reveal extensive ‘pro-stemness' network rewiring upon expression of two endogenous copies of the cancer-associated mutation *PIK3CA*^*H1047R*^.**

**What changes do you think could improve the professional lives of early-career scientists?**

I think a key challenge for early-career researchers is the stifling competition within academia, driven by a disconnect between assessment metrics for funding and what we as individuals consider key aspects of good science. In line with recent work from the Wellcome Trust, I think this challenge can be addressed through a major overhaul of current research culture, starting with the development of systems that actively reward the activities and behaviours that lead to good reproducible science. Together with more stable work contracts, this will unlock creativity and sustain a talented workforce.

“I think a key challenge for early-career researchers is the stifling competition within academia […]”

**What's next for you?**

I was incredibly fortunate to be awarded a Sir Henry Wellcome Postdoctoral Fellowship last year. This offers me 4 years to pursue my fascination with the context-dependent cellular effects of disease-causing *PIK3CA* mutations. I hope to address key unanswered questions in this area by harnessing the power of systems biology and improved disease model systems.
